# Overweight and obesity in Mexican children and adolescents during the last 25 years

**DOI:** 10.1038/nutd.2016.52

**Published:** 2017-03-13

**Authors:** S Hernández-Cordero, L Cuevas-Nasu, M C Morán-Ruán, I Méndez-Gómez Humarán, M A Ávila-Arcos, J A Rivera-Dommarco

**Affiliations:** 1 Centro de Investigación en Nutrición y Salud, Instituto Nacional de Salud Pública, Cuernavaca, Morelos, Mexico; 2 Centro de Investigación en Matemáticas A.C., Unidad Aguascalientes, Aguascalientes, México

## Abstract

**Background/Objective:**

The objective of the study was to provide current estimates of the prevalence and trends of overweight and obesity (OW+OB) in Mexican children and adolescents.

**Subjects/Methods:**

Body mass index objectively measured was analyzed for 37 147 children and adolescents aged 0–19 years obtained in 2012 as part of the National Health and Nutrition Survey (ENSANUT-2012), a nationally representative sample of the Mexican population. In addition, data from previous National Nutrition Surveys obtained in 1988, 1999 and 2006 were compared with analyze trends over a 24-year period (1988–2012) for children <5 years of age and adolescents and over a 13-year period (1999–2012) for school-age children. World Health Organization Child Growth Standard was used to define OW+OB.

**Results:**

In 2012, 33.5% of children <5 years of age (both sexes) were at risk of overweight or were overweight (OW); 32% and 36.9% of girls and boys 5–11 years of age were OW+OB, respectively, and 35.8% and 34.1% of female and male adolescents were OW+OB, respectively. Statistically significant trends were documented for all age groups during the study period. Overall change in the combined prevalence in preschool children was 6.3±1.0 percentage points (pp; *P*<0.001; 0.26 pp per year) in the last 24 years, showing the highest increase between 1988 and 1999, whereas for school-age girls (from 1999 to 2012) and adolescent females (from 1988 to 2012), OW+OB increased across all periods at a declining trend, with an overall change of 0.5 and 1.0 pp per year, respectively. Changes in the prevalence of OW+OB were highest among children and adolescents in the lowest quintile of the household living condition index.

**Conclusions:**

Prevalence of OW+OB among children and adolescents increased significantly during the last 13–24 years. The rate of increase has declined in the last 6 years in all age groups. Changes in prevalence of OW+OB presented here suggest that, in Mexico, the burden of obesity is shifting toward the groups with lower socioeconomic level.

## Introduction

Prevalence of overweight (OW) and obesity (OB) in all age groups has increased throughout most countries in recent decades, with childhood OB representing a public health challenge. Worldwide, in 2010 an estimated 42 million children were OW, and 35 million were living in developing countries.^1^

OB in childhood has immediate consequences on health including hyperlipidemia, hypertension and abnormal glucose tolerance as well as orthopedic, neurological, pulmonary, gastroenterological, endocrine and hepatic disorders, especially when OB is severe.^2, 3, 4^ Other consequences of OB are psychological effects and social stigmatization that obese youth face, which can produce serious consequences for emotional and physical health.^4, 5^ There is an established link between OB during childhood and its persistence into adolescence and adulthood.^2^ OW+OB are well-recognized risk factors for noncommunicable diseases in adults, such as hypertension, type 2 diabetes and cardiovascular diseases, among others.^3, 4^ It is expected that the increase of childhood OW+OB will be followed by the occurrence of chronic diseases at younger ages, with the associated disabilities and early death as well as increased expenses for families and country health systems.^4, 5^

The burden of childhood OB on the health system is also undeniable and cannot yet be fully estimated. Health problems will be seen in the next generation of adults as obese children today become obese adults.^5^

In Mexico, data from three national surveys conducted in 1988, 1999 and 2006, using the International Obesity Task Force (IOTF) classification system described the upward trends on OW and OB in school-age children and adolescents at the national level.^6^

The present paper provides the most recent prevalence estimates of OW+OB in Mexican children and adolescents aged 0–19 years using data from the 2012 Mexican National Health and Nutrition Survey and describes trends in OW and OB in the last 13–24 years, using the World Health Organization Child Growth Standard to define OW and OB.

## Materials and methods

### Study population and National Health and Nutrition Survey

Prevalence of OW+OB was calculated using data from the most recent National Health and Nutrition Survey–2012 (Encuesta Nacional de Salud y Nutrición or ENSANUT-2012 according to its acronym in Spanish). For trend analysis, data from previous National Health and Nutrition Surveys obtained in 1988, 1999, and 2006, along with the 2012 data, were used. ENSANUT-2012 was conducted from October 2011 to May 2012 by the National Institute of Public Health in Mexico. A nationally representative sample of the population was selected using a stratified, multistage probability sample design. The sample is representative at state, regional, and urban and rural levels. Information collected during the ENSANUT-2012 comprises anthropometric data (weight and height, among others). A more detailed description of ENSANUT methods has been published elsewhere.^7^ Informed consent was obtained from all participants and/or parents or primary caregiver (in case of young children), and the protocol was reviewed and approved by the Institutional Review Board of the National Institute of Public Health.

The study population for the present analyses includes children <19 years of age classified according to three different age groups: preschool children (<5 years of age), school-aged children (5–11 years) and adolescents (12–19 years). ENSANUT-2012 overall nonresponse rate was 8.7%. A total of 1.25% of examined children and adolescents had missing data for body mass index (BMI) either because of lack of one or both measurements, implausible data or in the case of pregnancy in adolescent girls. BMI plausible data were considered at a range of −5.0 and +5 s.d., eliminating all those with BMI values <10 or >38 for preschool children and school-age children and BMI <10 or >58 for adolescents. Excluded from the analyses were all cases where *z*-score for height and age was<−6 or >+6 s.d. For ENSANUT-2012, data for analyses were available for 10 658 preschool children, 16 351 school-age children and 13 992 adolescents (Table 1). Weight and height were measured with the same validated and standardized methods as well as same criteria were used to define plausible anthropometric data for the samples from previous surveys included in the analyses. For previous National Health and Nutrition Surveys of 1999 and 2006, nonresponse rate was 17.6%^8^ and 0.6%,^9^ respectively. Unfortunately, there is no information of 1988 survey's nonresponse rate.

### Definition of OW+OB for all nutritional surveys

All anthropometric information was measured using standardized techniques and equipment.^7^ BMI was calculated as weight (kg) divided by height (m) squared. We defined risk of overweight (RO), OW and OB among all age groups based on the World Health Organization (WHO) Child Growth Standard for preschool children^10^ and 2007 WHO growth reference for school-age children and adolescents.^11^ For preschool children, the RO category was defined as *z*-score of BMI<1 s.d. and 2 or less s.d. OW and OB was defined as *z*-score +2 s.d. For school-aged children and adolescents, OW was defined as *z*-score of BMI>1 s.d. and 2 or less s.d.; OB as BMI *z*-score >2 s.d.^9, 10^

### Other variables for analysis of ENSANUT-2012

Area of residence was classified according to the number of inhabitants, considering those areas with a population of 2500 or more as urban areas and those with <2500 persons as rural areas.

The Household Living Condition Index (HLCI) was used as a proxy of socioeconomic status and was constructed using the principal component analysis.^8^ The HLCI was constructed considering household characteristics (number of rooms, running water, WC and construction materials) as well as household amenities (washing machine, microwave, stove, television and so on). The first factor was used as the HLCI, explaining 40.5% of the variance. The index was further divided into quintiles of HLCI to be considered as categorical variables in the statistical analysis.

### Statistical analysis

For ENSANUT-2012 OW+OB prevalence analysis, we performed frequencies at a national level by age group, sex, area of residence (rural and qurban areas) and HLCI. Trends for OW+OB from the 1988, 1999, 2006 and 2012 surveys were calculated with a standardized proportions difference using the asymptotic normal distribution.^12^ School-age children of both sexes and male adolescents were not measured in 1988; thus, trends were estimated only from 1999 to 2012 in school-age children and for adolescents during the entire 1988–2012 period, but only for females.

To study trends in prevalence of OW and OB, descriptive analyses were performed using frequencies and their respective 95% confidence intervals (CIs) stratified at the national level for the four geographic regions, rural and urban areas, and HCLI categories. Multinomial logistic regression models with fixed effects^12^ were used to estimate differences among categories. Prevalence was further divided by age group blocks. Trends for OW+OB from the 1988, 1999 and 2006 surveys were calculated using the standardized difference between proportions.^12^ Due to the fact that the duration of the time periods between surveys differed, in order to compare the prevalence we present the changes as percentage points (pp) and pp per year.

Data were analyzed using the Statistical Program SPSS v. 15.0 with the complex sampling software (SPSS Inc., Chicago, IL, USA) and Stata v.12.1 (Stata Corp., College Station, TX, USA).

## Results

### Overweight and obesity: National Health and Nutrition Survey 2012

Overall among children 0–19 years of age, 28.8% presented either RO (preschool children) or OW, or OB in 2012. Table 2 shows detailed prevalence estimates of RO, OW+OB for preschool children and OW and OB for school-age children and adolescents. Results are presented by year of survey, sex (for school-age children and adolescents), area of residence (urban/rural) and HLCI. For comparison purposes we provide the OW+OB estimates using the IOTF definition^13^ as Supplementary Material, Supplementary Table 1.

#### Preschool children

Prevalence of RO and OW+OB was 23.8% (95% CI: 22.5, 25.1%) and 9.7% (95% CI: 8.9, 10.6%), respectively (Table 2). Differences in prevalence by sex were already observed at this early age, with a higher combined prevalence of RO and OW+OB in boys (35.2%) than in girls (31.8% *P*<0.001). This difference was mainly due to a higher prevalence of RO in boys (25.3%) than in girls (22.3% data not shown). There was no difference in RO or OW+OB either by area of residence or by HLCI.

#### School-age children

The combined prevalence of OW and OB in boys and girls in this age group was 34.4% (95% CI: 33.3, 35.6%) with the prevalence of OW being 19.8% (95% CI: 18.8, 20.9% Table 2). The combined prevalence of OW and OB was higher in boys (36.9%) than in girls (32.0%); *P*<0.001 (data not shown). The difference in the combined prevalence is due to the higher prevalence of OB among boys (17.4%, 95% CI: 16.0, 18.8%) than in girls (11.8%, 95% CI: 10.8, 12.8% *P*<0.001; Table 2).

The prevalence of both OW and OB was higher in boys and girls living in urban areas (Table 2). For girls, there was a trend for both OW and OB, increasing the prevalence as the HLCI increased. The highest prevalence was found in girls from the highest quintile (OW: HLCI lowest quintile: 14.9 (95% CI: 12.6, 17.5%)) vs HLCI highest quintile: 24.8% (95% CI: 21.1, 29.0% OB: HLCI lowest quintile: 7.9% (95% CI: 6.2, 9.9%)) vs HLCI highest quintile: 16.2 (95% CI: 13.6, 19.2% Table 2). For boys, the trend of increasing OW and OB was more pronounced for the latter (OW: HLCI lowest quintile 15.7; 95% CI: 13.4, 18.3%) vs HLCI highest quintile 20.3% (95% CI: 17.2, 23.9%); OB: HLCI lowest quintile 8.2% (95% CI: 6.3, 10.6%) vs HLCI highest quintile 22.7% (95% CI: 19.2, 26.8% Table 2).

#### Adolescents

Combined prevalence of OW and OB in this age group was 35.8% (95% CI: 34.0, 37.6%) and 34.1% (95% CI: 32.4, 35.8%) for female and male adolescents, respectively, being slightly higher in females (*P*=0.03; data not shown). The difference in combined prevalence by sex is mainly due to the higher prevalence of OW in females compared with that in males (23.7 vs 19.6, *P*<0.001, for females and males, respectively; Table 2). Prevalence of both OW and OB was higher in female and male adolescents living in urban areas, whereas the prevalence of OB was higher for adolescents (both male and female) in the highest quintile of the HLCI (OB prevalence for females: lowest HLCI quintile 8.2% (95% CI: 6.5, 10.2%), highest HLCI quintile 13.2 (95% CI: 10.9, 15.9%), males: lowest HLCI quintile 6.8% (4.8, 9.5%), highest HLCI quintile: 19.4% (16.4, 22.7% Table 2).

### Trends of OW and OB

#### Prevalence of OW and OB in female children and adolescents during the last 24 years

**Figure 1:**
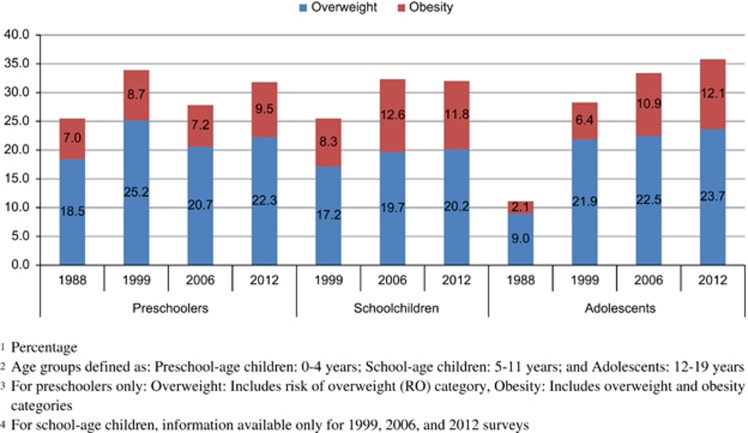
Prevalence of overweight and obesity in girls and female adolescents by age group and survey: 1988–2012.^1,2,3,4^

Overall trends of OW and OB for girls and female adolescents using the available information are presented in Figure 1. Complete information for the four surveys (1988, 1999, 2006 and 2012) is only available for preschool girls and female adolescents, whereas for school-aged girls there is information from only the 1999–2012 surveys. Prevalence of OW and OB increased in all age groups, with the highest rate of increase among female adolescents followed by school-aged girls.

For preschool girls, there has been a statistically significant increase in all years except between 1999 and 2006, years when the prevalence of RO and OW decreased (RO and OW combined prevalence change from 1999 to 2006: −6.1±1.0 pp-, *P*<0.001; or −0.87 pp per year). Overall change in the combined prevalence was 6.3±1.0 pp (*P*<0.001; 0.26 pp per year) in the last 24 years, showing the highest increase between 1988 and 1999 (combined prevalence change: 8.4±1.0 pp, *P*<0.001; 0.76 pp per year) compared with change from 2006 to 2012 (3.9±1.0 pp, *P*<0.001; 0.65 pp per year).

For school-aged girls, overall change in the combined prevalence of OW and OB from 1999 to 2012 was 6.2±0.8 pp (*P*<0.001; 0.5 pp per year), demonstrating the highest increase from 1999 to 2006 (6.5±0.8 pp, *P*<0.001; 0.9 pp per year), with no change from 2006 to 2012 (combined prevalence change: −0.3±0.7, *P*=0.65; −0.06 pp per year).

The combined prevalence of OW and OB in adolescent females increased (24.7±0.8 pp, *P*<0.001; 1.0 pp per year) from 1988 to 2012, showing the highest and marked increase between 1988 and 1999 (17.2±0.9 pp (*P*<0.001; 1.6 pp per year). The rate of increase began to slow down from 1999 onward; however, there has been a steady increase since then with statistical significance between each survey (combined prevalence change from 1999 to 2006: 5.1±0.9 pp, *P*<0.001; 0.7 pp per year); change from 2006 to 2012: 2.4±0.8 pp, *P*=0.002 (0.4 pp per year).

#### Trends by age group, HLCI and area of residence

**Preschool children (boys and girls; change from 1988 to 2012)**

Increase in the combined prevalence of RO and OW in preschool children occurred at all socioeconomic levels, without demonstrating any difference by HLCI level (data not shown).

**Figure 2:**
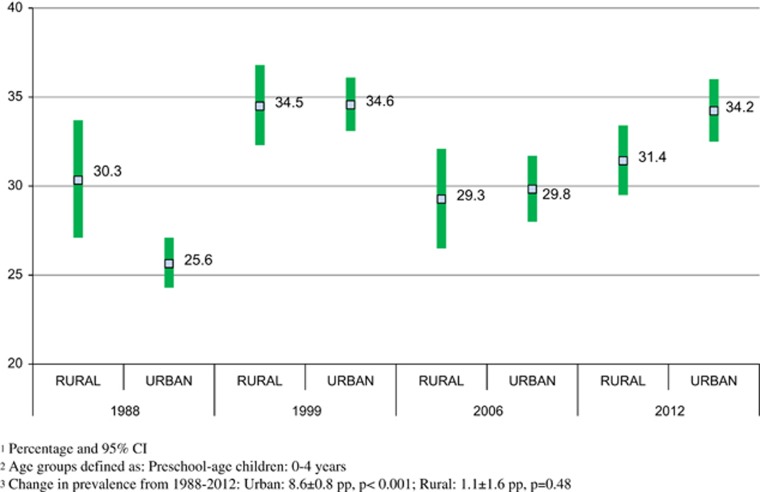
Combined prevalence of risk of overweight and overweight in preschool-age children (both sexes) by area of residence and year of survey: 1988–2012.^1, 2, 3^

When changes in the combined prevalence of RO and OW by area of residence were analyzed, the rate of increase was higher among children living in urban areas (1988 prevalence 25.6%, 95% CI: 24.3, 27.1% 2012 prevalence 34.2, 95% CI: 32.5, 36.0%); change 1988–2012: 8.6±0.8 pp, *P*<0.001 (0.36 pp per year) than in children living in rural areas (1988 prevalence 30.3, 95% CI: 27.1, 33.7% 2012 prevalence: 31.4, 95% CI: 29.5, 33.4%. Change from 1988 to 2012 was 1.1±1.6 pp, *P*=0.48 (0.05 pp per year; Figure 2).

**School-age children (change from 1999 to 2012)**

**Figure 3:**
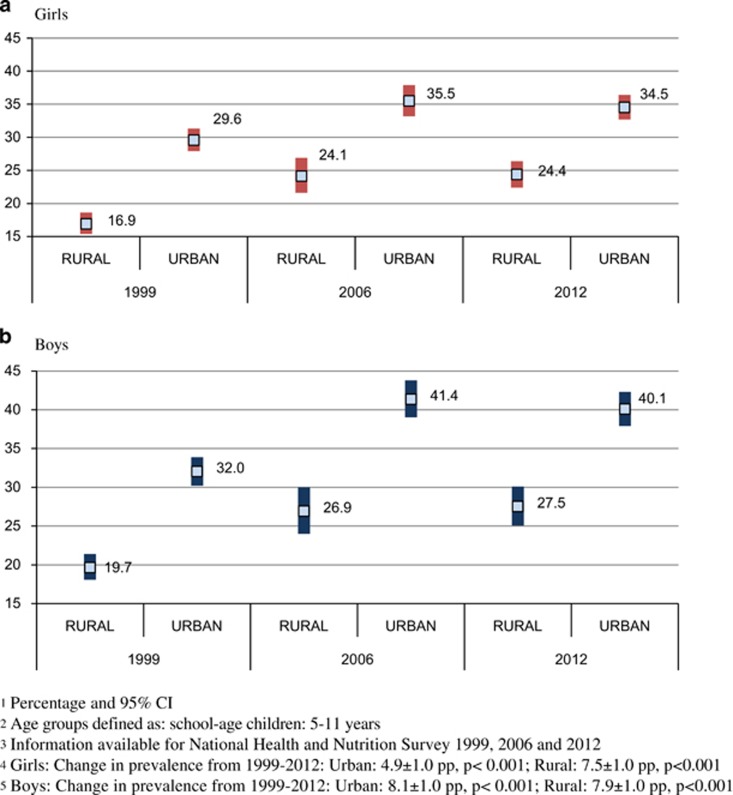
Combined prevalence of overweight and obesity in school-age girls (**a**) and boys (**b**) by area of residency and year of survey: 1999–2012.^1,2,3,4,5^ (**a**) Girls. (**b**) Boys.

**Figure 4:**
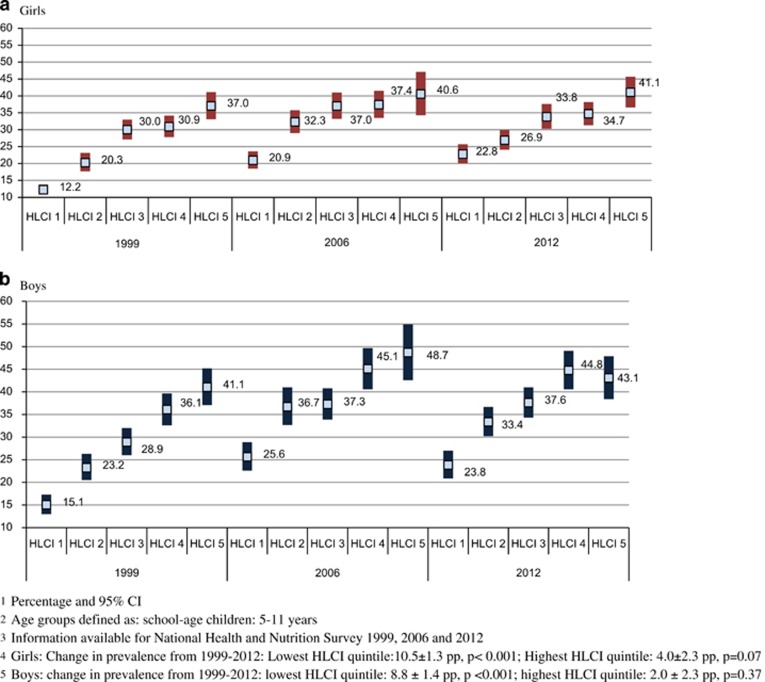
Combined prevalence of overweight and obesity in school-age girls (**a**) and boys (**b**) by household living conditions index (HLCI) and year of survey: 1999–2012.^1,2,3,4,5^ (**a**) Girls. (**b**) Boys.

Combined prevalence of OW and OB increased in both rural and urban areas from 1999 to 2012. However, the rate of increase was more pronounced in girls living in rural areas (change in prevalence 1999–2012: rural: 7.5±1.0 pp, *P*<0.001; 0.58 pp per year); urban: 4.9±1.0 pp, *P*<0.001 (0.38 pp per year; Figure 3). This trend was not seen among boys in whom the rate of change of combined prevalence of OW and OB was similar between area of residence (change in prevalence 1999–2012: rural: 7.9±1.0 pp, *P*<0.001 (0.60 pp per year); urban: 8.1±1.0 pp, *P*<0.001 (0.62 pp per year). For both boys and girls, the combined prevalence of OW and OB increased at a higher rate in the lower quintiles of the HLCI compared with the highest quintile (change of prevalence 1999–2012: girls—lowest HLCI quintile: 10.5±1.3 pp, *P*<0.001; highest HLCI quintile: 4.0±2.3 pp, *P*=0.07; boys—lowest HLCI quintile: 8.8±1.4 pp, *P*<0.001; highest HLCI quintile: 2.0, *P*=0.37; Figure 4).

**Female adolescents (change from 1988 to 2012)**

Combined prevalence of OW and OB increased 16±1.4 pp from 1988 to 1999 in rural areas (*P*=0.01) and 18.4±1.0 in urban areas. A smaller increase in the prevalence from 1999 to 2006 was seen in both areas (rural: 2.8±1.3, *P*=0.03; urban: 5.9±1.1, *P*=0.01). This rate of increase decreased during the last 6 years but was still on the rise among female adolescents living in urban areas (change in prevalence from 2006 to 2012: 2.6±1.0 pp, *P*=0.01; Supplementary Figure 1A).

For HLCI, prevalence of OW and OB increased >11 pp between 1988 and 1999 in all quintiles except the fourth, which showed an increase of 22.3±1.9 pp, *P*=0.01. From 1999, the rate of increase at all HLCI levels decreased and the prevalence for 2006 with respect to 1999 for Q1 and Q2 increased 3.8±1.6 pp (*P*=0.01) and 10.8±1.5 pp (*P*=0.01), respectively. Between 2006 and 2012, females in Q1 and Q4 showed an increase in the prevalence of OW and OB of 4.7±1.6 pp, *P*=0.003 and 3.8±1.8 pp, *P*=0.04, respectively (Supplementary Figure 1B).

**Male adolescents (change from 2006 to 2012)**

In male adolescents, no changes were observed in the combined prevalence of OW and OB between surveys from 2006 to 2012 (change in prevalence: 1.1±0.8 pp, *P*=0.16), with no difference by area of residence or HLCI (data not shown).

## Discussion

Prevalence of OW+OB in children and adolescents presented in this article are the most recent estimates in Mexico. Among children and adolescents <19 years of age the combined prevalence of OW (and RO for preschool children) and OB was as high as 28.8% by 2012. Combined prevalence of RO and OW+OB in children <5 years of age was 33.5% (RO: 23.8%, OW+OB: 9.7%) and for school-age children and adolescents was 36.9% (OW: 19.5%, OB: 17.4%) and 35.8% (OW: 23.7%, OB: 12.1%), respectively. By 2012, the highest prevalence was seen among children and adolescents living in urban areas and those from the highest socioeconomic level. In the last 13–24 years, the prevalence of OW and OB has increased at the highest rate among female adolescents followed by school-aged girls. Increase in OW and OB has been more pronounced among those children from the lowest socioeconomic level in all age groups except for preschool children and those from urban areas for preschool children and adolescents, whereas for school-aged girls, the increase has been higher among those living in rural areas.

When comparing prevalence from other countries using the WHO classification systems (WHO Child Growth Standard for preschool children^10^ and WHO growth reference for school-age children and adolescents WHO 2007,^11^ childhood prevalence of OW and OB in Mexican children is among the highest. A longitudinal study carried out in Cuba demonstrated that the combined prevalence of RO and OW in children <5 years of age was 17.3% in 2011^14^ and for Colombian preschoolers was 25.2% in 2005 according to a recent systematic review.^15^ Prevalence of OW and OB in school-age children in Brazilian boys was 34.8% in 2009 and 18.9% in 2010 in Colombian children (both sexes), lower than the prevalence in Mexico during the same time (2012), but the prevalence of OW and OB in Chilean children (both sexes) in 1997 was 37.7%, much higher than the prevalence shown in Mexican boys (28.2%) and girls (25.5%) during the same year (1999). Prevalence of OW+OB in adolescents was relatively low in Colombian adolescents in 2010 (16.7%) compared with Chile in 2005 (31.0%), and Mexican males (34.1%) and females (35.8%) in 2012.^15^

In 2012, the combined prevalence of OW and OB was higher in preschool and school-age boys than in girls, whereas in adolescents, such prevalence was higher among females than males. Similar sex differences have been reported by Rivera *et al.*^15^ who found that in those studies reporting data stratified by sex, prevalence of OW and OB differed by sex, but the differences varied across age groups. As in our study, in school-age children, more boys were identified as OW or OB than girls in Brazil. On the other hand, in adolescents, unlike in Mexico, the prevalence of OW and OB was higher among adolescent Brazilian males than in their female counterparts.

Results of the latest Mexican nutritional survey (2012) show that the combined prevalence of OW and OB in school-aged children and adolescents was higher among those in the highest quintile of the HLCI, a wealth indicator, whereas in preschool children there were no differences according to socioeconomic status indicator. Clustering of OW and OB by socioeconomic status varies depending upon the study population. There is growing evidence that the distribution of OW and OB varies by socioeconomic status and area of residence, which depends on the economic development of the countries.^16^ In some countries such as Russia,^17^ China,^17^ Croatia,^18^ Estonia,^18^ Latvia^18^ and Botswana,^19^ prevalence of OW and OB was higher among more affluent families, similar to our results. In other countries such as the USA^16, 20^ and Spain,^21^ the highest rates of OW and OB occur among the most disadvantaged groups.

An important finding of our study is that although the prevalence of OW and OB is still higher in the population with higher socioeconomic status, the rate of increase of such prevalence in the last 24 years has been higher in school-aged children and adolescents from the lowest quintile of socioeconomic status. Some explanations for this rapid increase among the poorer strata is supported by the increasing evidence that low-income families tend to consume inexpensive sources of calories due to the relative cost of nutritious foods both in money and preparation time. The source of these calories is usually from energy-dense foods with high-fat content and sugar as well as with poor nutritional quality (low content of vitamins and minerals).^20^ A recent analysis in Mexico shows that patterns of intake are different depending on the level of income. The results indicate that the cost per calorie (defined as the amount of money allocated to consume 1 calorie) decreased between 1992 and 2010. Households with lower-income levels make consumption decisions that allow them to obtain a higher level of calories at a lower price, even if this represents a lower dietary quality.^22^ The lower the socioeconomic level, the greater the percentage of purchase and consumption of high-energy-dense foods as well as the lower the purchase and consumption of low-energy-dense foods (mainly fruit and vegetables and low-fat/-sugar foods).^22^ This phenomena has been reported by others, indicating that there is a negative association between income and dietary quality. Thus, as incomes decrease, nutrient-poor energy-dense foods become the best way to provide daily calories at an affordable cost, to the detriment of healthier foods (such as fruits and vegetables), which are more expensive.^23^

There is little information in terms of changes in physical activity among children and adolescents in the last 24 years. There are few studies in Mexican preschool^24^ and school-aged children,^25^ and adolescents.^26^ These studies indicate that low physical activity level and sedentary behavior are common among these age groups. In addition, there is evidence that some of the determinants of low physical activity and sedentary behavior such as urbanization, use of innovation in electronic media (computers, televisions) among others have increased in the Mexican population during the last 20 years.^27^

Another relevant result of this study is the trend of the combined prevalence of OW and OB among preschool girls shown to decrease between 1999 and 2006. As reported previously by Rivera *et al.*,^28^ the observed decrease in prevalence for this group may be the result of the decline in the prevalence of low height for age (stunting) observed during the same time period, which was about 0.86 pp per year.

Finally, it is worth mentioning that among school-age girls there was an apparent plateau in the combined prevalence of OW and OB from 2006 to 2012. This trend has been reported in developed countries like the USA where the prevalence shown in some age groups such as school-age children and adolescents appears to be leveling off^29^ or Denmark where comparison of prevalence from 1998 to 2011 showed that the prevalence rates of OW and OB among Danish infants, children and adolescents were largely still at a plateau with tendencies for a decline among children and adolescents.^30^ Results in the same direction were previously reported in a review of prevalence of OW and OB in nine countries (Australia, China, England, France, The Netherlands, New Zealand, Sweden, Switzerland and USA), where prevalence of OW and OB was stabilizing.^31^ It may be too early to conclude that this is happening with the prevalence of OW and OB in school-age children. Follow-up in regard to this trend with further National Nutrition Surveys will be useful to answer this question. In addition, although Mexico is demonstrating a plateau in OW and OB in school-age children, a public health crisis remains as a result of the high prevalence of childhood OB.

This paper describes the most recent trends of childhood OB in Mexico. It includes information from different age groups during childhood from four nationally representative surveys during the last 25 years. Mexico is one of the few countries in the Latin American and Caribbean region with repeated National Nutrition Surveys across time, allowing us to explore trends.

Another strength of this study is that we used BMI as the indicator of body fatness. Although the use of BMI as a measure of health risk related to body fat has been criticized, especially in children,^32^ there is evidence that BMI in children is highly correlated with body fat mass and widely used as a valid indirect measurement of adiposity in children. There has been an increased number of growth references expressed as a function of age and sex.^31, 32^

An additional strength of the study is the use of the WHO Child Growth Standard for preschool children^10^ and the 2007 WHO growth reference for school-age children and adolescents^11^ with their respective definitions of OW and OB, thus facilitating comparison with data from other countries.

One limitation of our study is that not all age groups (lack of information in school-age children on the 1988 national survey) and sometimes not both sexes, particularly boys and adolescent males (school-age boys and male adolescents were not measured in 1988 and 2006), have complete information for the analysis of prevalence and trends in the last 25 years, limiting our conclusions for those groups for trends. However, we studied complete trends during the last 25 years for girls and indicated, when necessary, the time frame of trends in cases where information on both sexes was included.

## Conclusions

Prevalence of childhood OB in Mexico is one of the highest worldwide. Even though the prevalence shown here indicates a higher prevalence of OW and OB among those in urban areas and those from more affluent families, changes in prevalence of OW and OB presented here suggest that, as in other countries, in Mexico the burden of OB is shifting toward groups with a lower socioeconomic level.

## Figures and Tables

**Table 1:** Sample size and descriptive characteristics of children and adolescents by age group (National Health and Nutrition Surveys 1988, 1999, 2006 and 2012)^a^

*Characteristic*	*Preschoolers (0*–*4 years)*			*School-age children (5*–*11 years)*			*Adolescents (12*–*19 years)*		
	n *(sample)*	n *(thousands)*	*%*	n *(sample)*	n *(thousands)*	*%*	n *(sample)*	n *(thousands)*	*%*
*1988*									
Total	6794	8268.1	100				4917	5776.5	100
Sex									
Male	3455	4216.8	51.0				–	–	–
Female	3339	4051.4	49.0				4917	5776.5	100
Area									
Urban	5702	6518.8	78.8				4248	4770.8	82.6
Rural	1092	1749.3	21.2				669	1005.7	17.4
HLCI-Q									
Q1	1189	1847.0	22.3				828	1104.8	20.0
Q2	1228	1452.7	17.6				914	1139.8	20.6
Q3	1191	1280.7	15.5				1204	1330.7	24.1
Q4	1116	1203.6	14.6				832	893.3	16.2
Q5	1134	1270.8	15.4				939	1058.1	19.1
									
*1999*									
Total	7473	10125.9	100	11 274	15405.7	100	5070	7559.0	100
Sex									
Male	3786	5083.1	50.2	5568	7546.7	49.0	–	–	–
Female	3687	5042.9	49.8	5706	7859.0	51.0	5070	7559.0	100
Area									
Urban	4406	7138.8	70.5	6336	10596.0	68.8	3015	5410.9	71.6
Rural	3067	2987.1	29.5	4938	4809.6	31.2	2055	2148.1	28.4
HLCI-Q									
Q1	1850	2240.5	22.1	2836	3410.0	22.1	1088	1404.6	18.6
Q2	1529	1896.3	18.7	2166	2767.3	18.0	961	1284.8	17.0
Q3	1633	2103.4	20.8	2390	3149.7	20.4	1096	1551.8	20.5
Q4	1242	1811.5	17.9	2050	2842.3	18.4	930	1340.9	17.7
Q5	1020	1839.5	18.2	1552	2904.6	18.9	875	1809.8	23.9
									
*2006*									
Total	7697	9400.1	100	15 045	15749.4	100	14 445	18320.1	100
Sex									
Male	3945	4765.7	50.7	7518	7834.5	49.7	7088	9163.3	50.0
Female	3752	4634.3	49.3	7527	7914.9	50.3	7357	9156.7	50.0
Area									
Urban	5366	6933.8	73.8	10 112	11340.6	72.0	10 146	13542.0	73.9
Rural	2331	2466.2	26.2	4933	4408.8	28.0	4299	4778.1	26.1
HLCI-Q									
Q1	1923	2317.3	24.7	3953	4068.2	25.8	3115	3952.9	21.6
Q2	2000	2264.5	24.1	3607	3534.9	22.4	3308	3797.3	20.7
Q3	1616	1841.7	19.6	3071	2908.9	18.5	3001	3587.1	19.6
Q4	1302	1740.5	18.5	2586	2913.0	18.5	2743	3565.8	19.5
Q5	829	1208.0	12.9	1773	2276.4	14.5	2221	3341.8	18.2
									
*2012*									
Total	10 658	10785.1	100	16 351	16444.1	100	13 992	18102.8	100
Sex									
Male	5314	5418.9	50.2	8195	8327.4	50.6	7041	9232.1	51.0
Female	5344	5366.2	49.8	8156	8116.7	49.4	6951	8870.7	49.0
Area									
Urban	6569	8036.8	74.5	10 126	12262.9	74.6	9017	13659	75.5
Rural	4089	2748.3	25.5	6225	4181.3	25.4	4975	4443.8	24.5
HLCI-Q									
Q1	2646	2163.4	20.1	3827	3146.2	19.1	2827	3018.0	16.7
Q2	2413	2115.6	19.6	3630	3124.5	19.0	2860	3268.3	18.1
Q3	2223	2176.0	20.2	3347	3252.7	19.8	2899	3541.5	19.6
Q4	1947	2342.9	21.7	3088	3519.3	21.4	2763	3923.6	21.7
Q5	1429	1987.2	18.4	2459	3401.5	20.7	2643	4351.3	24.0

Abbreviations: HLCI, Household Living Condition Index; Q, Quintile.

a Age groups defined as preschoolers: 0–4 years; school-age children: 5–11 years; and adolescents 12–19 years.

**Table 2:** Prevalence of risk of overweight, overweight and obesity by age group, sex, area of residence HLCI (Surveys 1988, 1999, 2006 and 2012)^a^^,^ b ^,^ c ^,^ d ^,^ e

*Variable*		*1988*						*1999*						*2006*						*2012*					
		*Overweight*			*Obesity*			*Overweight*			*Obesity*			*Overweight*			*Obesity*			*Overweight*			*Obesity*		
		n *(thousands)*	*%*	*95% CI*	n *(thousands)*	*%*	*95% CI*	n *(thousands)*	*%*	*95% CI*	n *(thousands)*	*%*	*95% CI*	n *(thousands)*	*%*	*95% CI*	n *(thousands)*	*%*	*95% CI*	n *(thousands)*	*%*	*95% CI*	n *(thousands)*	*%*	*95% CI*
Preschool children	Boys and girls																								
National	Total	1553.1	18.8	(17.7, 19.9)	649.1	7.9	(7.0, 8.8)	2603.5	25.7	(24.7, 26.8)	894.2	8.8	(8.1, 9.6)	2006.7	21.3	(19.9, 22.9)	783.3	8.3	(7.5, 9.3)	2566.7	23.8	(22.5, 25.1)	1047.5	9.7	(8.9, 10.6)
Area	Urban	1216.5	18.7	(17.5, 19.9)	455.1	7.0	(6.2, 7.9)	1820.9	25.5	(24.3, 26.8)	646.4	9.1	(8.2, 10.0)	1469.4	21.2	(19.5, 23.0)	598.8	8.6	(7.6, 9.8)	1929.2	24.0	(22.4, 25.7)	821.3	10.2	(9.2, 11.4)
	Rural	336.6	19.2	(16.5, 22.3)	194.1	11.1	(8.5, 14.3)	782.5	26.2	(24.3, 28.2)	247.7	8.3	(7.3, 9.4)	537.3	21.8	(19.4, 24.4)	184.4	7.5	(6.0, 9.2)	637.5	23.2	(21.4, 25.1)	226.1	8.2	(7.1, 9.6)
HLCI	Q1	383.5	20.8	(18.2, 23.5)	147.2	8.0	(5.7, 11.0)	588.4	26.3	(24.2, 28.4)	175.2	7.8	(6.6, 9.2)	536.2	23.1	(20.5, 26.0)	161.3	7.0	(5.4, 8.9)	533.6	24.7	(22.3, 27.1)	206.9	9.6	(7.8, 11.7)
	Q2	256.2	17.6	(15.1, 20.5)	110.3	7.6	(6.1, 9.5)	483.6	25.5	(23.3, 27.8)	170.9	9.0	(7.5, 10.8)	461.3	20.4	(17.7, 23.3)	200.2	8.8	(7.3, 10.7)	485.8	23.0	(20.5, 25.6)	174.7	8.3	(6.7, 10.1)
	Q3	224.8	17.6	(15.0, 20.4)	68.5	5.3	(4.0, 7.1)	509.7	24.2	(22.3, 26.3)	201.7	9.6	(8.1, 11.3)	374.7	20.3	(17.1, 24.0)	158.1	8.6	(6.9, 10.6)	497.7	22.9	(20.4, 25.6)	243.2	11.2	(9.4, 13.3)
	Q4	225.4	18.7	(16.0, 21.8)	78.7	6.5	(5.0, 8.5)	486.3	26.8	(24.4, 29.5)	163.1	9.0	(7.2, 11.2)	382.4	22.0	(18.1, 26.4)	162.4	9.3	(7.2, 12.0)	554.4	23.7	(20.7, 26.9)	194.3	8.3	(6.8, 10.1)
	Q5	243.9	19.2	(16.5, 22.2)	122.4	9.6	(7.7, 12.0)	485.0	26.4	(23.4, 29.6)	167.1	9.1	(7.5, 10.9)	247.6	20.5	(16.8, 24.7)	98.2	8.1	(6.1, 10.8)	495.2	24.9	(21.5, 28.7)	228.4	11.5	(9.2, 14.3)
																									
School-age children	Girls																								
National	Total							1354.0	17.2	(16.2, 18.3)	653.8	8.3	(7.5, 9.2)	1557.3	19.7	(18.3, 21.2)	999.7	12.6	(11.2, 14.2)	1638.1	20.2	(18.8, 21.6)	957.7	11.8	(10.8, 12.8)
Area	Urban							1035.5	19.3	(17.9, 20.8)	551.3	10.3	(9.1, 11.5)	1166.6	20.5	(18.7, 22.4)	855.3	15.0	(13.2, 17.0)	1312.3	21.6	(19.9, 23.4)	788.1	13.0	(11.7, 14.3)
	Rural							318.4	12.8	(11.6, 14.0)	102.5	4.1	(3.3, 5.1)	390.7	17.6	(15.4, 20.1)	144.4	6.5	(5.3, 8.0)	325.8	16.0	(14.3, 17.9)	169.5	8.3	(7.1, 9.7)
HLCI	Q1							181.3	10.4	(9.2, 11.8)	32.1	1.8	(1.3, 2.7)	315.8	15.2	(13.1, 17.4)	120.7	5.8	(4.5, 7.4)	228.7	14.9	(12.6, 17.5)	120.7	7.9	(6.2, 9.9)
	Q2							212.7	15.0	(12.7, 17.7)	73.8	5.2	(3.9, 6.9)	336.9	18.9	(16.3, 21.8)	238.1	13.4	(10.8, 16.5)	287.8	18.0	(15.4, 20.8)	143.1	8.9	(7.2, 11.0)
	Q3							313.5	19.7	(17.4, 22.3)	164.1	10.3	(8.7, 12.2)	316.0	21.9	(18.6, 25.6)	217.9	15.1	(12.8, 17.8)	356.4	22.1	(19, 25.7)	188.0	11.7	(9.8, 13.9)
	Q4							287.0	20.5	(17.8, 23.4)	146.1	10.4	(8.5, 12.8)	322.6	23.6	(20.2, 27.3)	189.5	13.8	(11.4, 16.7)	371.8	20.8	(17.8, 24.2)	248.8	13.9	(11.5, 16.8)
	Q5							339.2	21.9	(18.8, 25.4)	233.7	15.1	(12.5, 18.2)	260.5	21.5	(16.8, 27)	231.8	19.1	(14.2, 25.2)	393.4	24.8	(21.1, 29)	257.1	16.2	(13.6, 19.2)
																									
	Boys																								
National	Total							1406.1	18.6	(17.4, 19.9)	724.6	9.6	(8.7, 10.5)	1626.1	20.8	(19.1, 22.5)	1297.9	16.6	(15.2, 18.1)	1622.0	19.5	(18.1, 21.0)	1447.1	17.4	(16.0, 18.8)
Area	Urban							1050.7	20.1	(18.5, 21.8)	624.3	11.9	(10.8, 13.2)	1241.7	22.0	(19.9, 24.3)	1092.3	19.4	(17.5, 21.3)	1271.9	20.6	(18.8, 22.5)	1205.3	19.5	(17.8, 21.4)
	Rural							355.4	15.3	(14.0, 16.8)	100.4	4.3	(3.5, 5.3)	384.3	17.5	(15.3, 20.0)	205.5	9.4	(7.8, 11.3)	350.1	16.3	(14.4, 18.3)	241.8	11.2	(9.6, 13.1)
HLCI	Q1							215.4	12.9	(11.3, 14.8)	35.5	2.1	(1.4, 3.2)	358.8	18.1	(15.4, 21.1)	150.0	7.6	(6.1, 9.3)	252.8	15.7	(13.4, 18.3)	131.8	8.2	(6.3, 10.6)
	Q2							229.4	17.0	(14.6, 19.6)	85.0	6.3	(4.9, 8.0)	388.3	22.1	(18.7, 26.0)	256.3	14.6	(12.3, 17.3)	271.5	17.8	(15.4, 20.5)	236.6	15.5	(12.8, 18.7)
	Q3							291.2	18.7	(15.9, 21.8)	159.2	10.2	(8.0, 12.9)	276.3	18.8	(16.2, 21.8)	270.3	18.4	(16.0, 21.2)	323.5	19.7	(16.9, 22.8)	294.4	17.9	(15.4, 20.8)
	Q4							330.0	22.9	(19.9, 26.2)	190.0	13.2	(11.1, 15.6)	306.5	19.9	(16.8, 23.3)	390.0	25.3	(20.9, 30.2)	404.3	23.3	(20.0, 27.1)	370.9	21.4	(18.6, 24.6)
	Q5							318.3	23.4	(20.1, 27.2)	239.3	17.6	(15.0, 20.6)	290.6	27.4	(21.2, 34.5)	227.0	21.4	(17.4, 26.0)	369.9	20.3	(17.2, 23.9)	413.4	22.7	(19.2, 26.8)
	Boys and girls							2760.1	17.9	(17.1, 18.7)	1378.4	8.9	(8.3, 9.6)	3183.3	20.2	(19.1, 21.4)	2297.6	14.6	(13.5, 15.7)	3260.1	19.8	(18.8, 20.9)	2404.8	14.6	(13.7, 15.6)
Adolescents	Women																								
National	Total	519.1	9.0	(8.1, 10.0)	120.9	2.1	(1.6, 2.7)	1652.0	21.9	(20.6, 23.2)	483.9	6.4	(5.7, 7.1)	2063.3	22.5	(21.1, 24.0)	995.4	10.9	(9.7, 12.2)	2103.6	23.7	(22.1, 25.5)	1074.1	12.1	(10.9, 13.4)
Area	Urban	452.5	9.5	(8.5, 10.6)	112.8	2.4	(1.8, 3.0)	1245.0	23.0	(21.1, 25.1)	388.9	7.2	(5.9, 8.7)	1568.5	23.5	(21.8, 25.3)	839.3	12.6	(11.0, 14.3)	1642.0	24.9	(22.8, 27.1)	906.9	13.7	(12.2, 15.4)
	Rural	66.6	6.6	(4.7, 9.3)	8.1	0.8	(0.3, 1.9)	407.0	19.0	(17.1, 20.9)	95.0	4.4	(3.4, 5.8)	494.7	19.9	(17.4, 22.7)	156.1	6.3	(5.0, 7.9)	461.5	20.3	(18.1, 22.7)	167.2	7.4	(6.1, 8.8)
HLCI	Q1	95.6	8.7	(6.6, 11.2)	2.0	0.2	(0.1, 0.6)	261.6	18.6	(16.1, 21.4)	25.4	1.8	(1.1, 3.0)	357.8	17.8	(15.3, 20.7)	128.9	6.4	(4.9, 8.4)	304.4	20.8	(17.8, 24.2)	119.2	8.2	(6.5, 10.2)
	Q2	99.9	8.8	(6.9, 11.2)	26.3	2.3	(1.4, 3.8)	285.1	22.2	(19.0, 25.8)	61.6	4.8	(3.4, 6.8)	500.2	25.5	(22.6, 28.6)	243.0	12.4	(9.8, 15.5)	411.2	24.4	(20.8, 28.3)	175.2	10.4	(8.3, 12.9)
	Q3	111.3	8.4	(6.6, 10.5)	31.1	2.3	(1.5, 3.6)	316.9	20.4	(17.5, 23.7)	122.9	7.9	(5.8, 10.6)	438.6	23.7	(20.3, 27.5)	229.8	12.4	(9.8, 15.7)	411.6	23.8	(20.6, 27.3)	236.8	13.7	(10.9, 17.1)
	Q4	85.6	9.6	(7.5, 12.2)	19.3	2.2	(1.2, 3.8)	311.0	23.2	(20.0, 26.8)	145.6	10.9	(7.9, 14.7)	410.3	23.6	(20.1, 27.4)	199.4	11.5	(8.8, 14.7)	457.2	24.8	(21.1, 28.8)	258.9	14.0	(11.7, 16.7)
	Q5	98.3	9.3	(7.3, 11.8)	36.6	3.5	(2.2, 5.5)	442.9	24.5	(20.5, 29.0)	123.8	6.8	(5.1, 9.0)	350.9	22.6	(18.5, 27.4)	189.6	12.2	(9.6, 15.4)	519.1	24.2	(21.0, 27.8)	284.0	13.2	(10.9, 15.9)
																									
	Males																								
National	Total													1834.8	20.0	(18.5, 21.6)	1187.7	13.0	(11.3, 14.8)	1809.4	19.6	(18.2, 21.1)	1338.1	14.5	(13.3, 15.8)
Area	Urban													1431.6	20.8	(19.0, 22.8)	994.3	14.5	(12.4, 16.8)	1436.6	20.3	(18.6, 22.2)	1151.7	16.3	(14.8, 17.9)
	Rural													403.2	17.6	(15.5, 19.9)	193.4	8.4	(6.6, 10.7)	372.8	17.2	(15.3, 19.3)	186.4	8.6	(7.2, 10.3)
HLCI	Q1													327.7	16.8	(14.3, 19.8)	105.8	5.4	(4.0, 7.4)	249.0	16.0	(13.3, 19.1)	106.0	6.8	(4.8, 9.5)
	Q2													358.0	19.5	(16.8, 22.6)	190.9	10.4	(8.5, 12.7)	288.4	18.3	(15.4, 21.6)	173.3	11.0	(8.6, 13.8)
	Q3													360.5	20.7	(17.2, 24.7)	255.3	14.7	(11.7, 18.2)	361.1	19.9	(17.0, 23.2)	223.3	12.3	(10.3, 14.7)
	Q4													394.8	21.6	(18.2, 25.5)	301.3	16.5	(13.8, 19.6)	430.0	20.7	(17.9, 23.7)	408.2	19.6	(16.6, 23.1)
	Q5													391.7	21.9	(18.1, 26.2)	332.8	18.6	(12.8, 26.2)	480.8	21.8	(18.4, 25.6)	427.3	19.4	(16.4, 22.7)
	Males and females													3898.0	21.3	(20.2, 22.4)	2183.1	11.9	(10.9, 13.0)	3912.9	21.6	(20.5, 22.8)	2412.2	13.3	(12.5, 14.2)

Abbreviations: BMI, body mass index; CI, confidence interval; HLCI, Household Living Condition Index; Q, Quintile; WHO, World Health Organization.

a Percentage and 95% CI.

b Age groups defined as: preschoolers: 0–4 years; school-age children: 5–11 years; and adolescents: 12–19 years.

c For preschoolers, overweight category refers to risk of overweight; obesity category includes overweight and obesity.

d For preschoolers at risk of overweight: *z*-score of BMI/age >1 s.d. (WHO); overweight and obesity: *z*-score of BMI/age ⩾2 s.d. (WHO). For school-aged children and adolescents: overweight *z*-score of BMI/age >1 s.d. and ⩽2 s.d. (WHO); obesity: *z*-score of BMI/age >2 s.d. (WHO).

e No data available for school-age children in 1988.
