# Supplementary material for: Overweight and obesity in Mexican children and adolescents during the last 25 years
Source: Nutr Diabetes. 2017 Mar 13;7(3):e247–. doi: 10.1038/nutd.2016.52 (PMC5380891; doi:10.1038/nutd.2016.52)
Supplement: Supplementary Table 1 [file nutd201652x1.pdf]

Supplementary table 1. Combined prevalence of risk of overweight, overweight and obesity by age group, sex, area of residency and household living conditions index (HLCI). Surveys 1988, 1999, 2006 and 2012. IOTF [1,2,3,4,5]

		1988						1999						2006						2012					
		Overweight*			Obesity			Overweight*			Obesity			Overweight*			Obesity			Overweight*			Obesity		
**VARIABLE**		n (Thousands)	%	CI95%	n (Thousands)	%	CI95%	n (Thousands)	%	CI95%	n (Thousands)	%	CI95%	n (Thousands)	%	CI95%	n (Thousands)	%	CI95%	n (Thousands)	%	CI95%	n (Thousands)	%	CI95%
**Preschool-age children**	***Boys and girls***																								
**National**	**Total**	**501.3**	**6.1**	**(5.3 , 6.9)**	**176.9**	**2.1**	**(1.7 , 2.6)**	**940.0**	**9.3**	**(8.6 , 9.9)**	**215.8**	**2.1**	**(1.8 , 2.5)**	**765.8**	**8.1**	**(7.1 , 9.3)**	**267.4**	**2.8**	**(2.3 , 3.3)**	**880.8**	**8.2**	**(7.3 , 9.0)**	**310.9**	**2.9**	**(2.4 , 3.4)**
Area	Urban	367.4	5.6	(4.9 , 6.4)	122.0	1.9	(1.4 , 2.3)	670.1	9.4	(8.5 , 10.2)	163.2	2.3	(1.8 , 2.7)	513.7	7.4	(6.2 , 8.7)	219.0	3.2	(2.5 , 3.8)	674.6	8.4	(7.4 , 9.4)	258.5	3.2	(2.6 , 3.9)
	Rural	133.9	7.7	(5.5 , 10.3)	54.9	3.1	(1.9 , 4.9)	269.9	9.0	(8.0 , 10.1)	52.6	1.8	(1.3 , 2.2)	252.2	10.2	(8.2 , 12.6)	48.4	2.0	(1.4 , 2.6)	206.2	7.5	(6.4 , 8.6)	52.4	1.9	(1.4 , 2.4)
HLCI	Q1	116.2	6.3	(4.6 , 8.4)	43.7	2.4	(1.3 , 4.0)	210.4	9.4	(8.4 , 10.4)	32.1	1.4	(1.0 , 2.0)	214.4	9.3	(7.2 , 11.6)	42.0	1.8	(1.2 , 2.6)	220.6	10.2	(8.4 , 12.3)	40.6	1.9	(1.2 , 2.8)
	Q2	70.6	4.9	(3.6 , 6.3)	30.1	2.1	(1.2 , 3.3)	201.3	10.6	(9.1 , 12.2)	34.4	1.8	(1.1 , 2.7)	165.2	7.3	(5.8 , 9.0)	64.1	2.8	(2.0 , 3.9)	134.4	6.4	(5.1 , 7.7)	44.7	2.1	(1.2 , 3.4)
	Q3	82.4	6.4	(4.8 , 8.5)	18.5	1.4	(0.8 , 2.5)	168.4	8.0	(6.6 , 9.6)	44.8	2.1	(1.6 , 2.8)	141.3	7.7	(5.9 , 9.7)	53.0	2.9	(1.9 , 4.1)	167.7	7.7	(6.3 , 9.3)	84.0	3.9	(2.6 , 5.5)
	Q4	55.1	4.6	(3.1 , 6.5)	21.1	1.8	(1.0 , 2.9)	166.1	9.2	(7.5 , 11.1)	42.1	2.3	(1.5 , 3.5)	133.5	7.7	(4.8 , 12.0)	75.4	4.3	(2.8 , 6.4)	160.6	6.9	(5.4 , 8.5)	60.8	2.6	(1.7 , 3.8)
	Q5	79.7	6.3	(4.7 , 8.3)	36.7	2.9	(1.8 , 4.3)	176.7	9.6	(7.8 , 11.6)	54.5	3.0	(2.1 , 4.1)	106.6	8.8	(6.3 , 12.1)	32.5	2.7	(1.6 , 4.3)	197.5	9.9	(7.5 , 12.9)	80.9	4.1	(2.6 , 6.2)
**School-age children**	***Girls***																								
**National**	**Total**							**1128.5**	**14.3**	**(13.2 , 15.3)**	**455.2**	**5.8**	**(5.0 , 6.5)**	**1428.5**	**18.0**	**(16.6 , 19.5)**	**678.0**	**8.6**	**(7.4 , 9.7)**	**1493.3**	**18.3**	**(17.0 , 19.7)**	**651.3**	**8.0**	**(7.1 , 8.8)**
Area	Urban							884.3	16.4	(14.9 , 17.8)	385.5	7.1	(6.1 , 8.2)	1095.2	19.2	(17.4 , 21.1)	607.3	10.7	(9.2 , 12.2)	1223.6	20.0	(18.3 , 21.7)	535.0	8.8	(7.7 , 9.8)
	Rural							244.2	9.8	(8.7 , 10.8)	69.7	2.8	(2.2 , 3.4)	333.2	15.0	(12.7 , 17.6)	70.7	3.2	(2.4 , 4.0)	269.7	13.2	(11.5 , 15.0)	116.4	5.7	(4.7 , 6.8)
HLCI	Q1							129.4	7.4	(6.2 , 8.7)	20.3	1.2	(0.8 , 1.6)	239.6	11.5	(9.5 , 13.7)	81.9	3.9	(2.8 , 5.3)	184.6	12.0	(9.9 , 14.4)	82.4	5.4	(4.0 , 7.0)
	Q2							165.2	11.6	(9.5 , 14.1)	56.6	4.0	(2.7 , 5.7)	295.4	16.6	(14.0 , 19.4)	172.1	9.7	(7.2 , 12.7)	261.4	16.2	(13.6 , 19.0)	86.8	5.4	(4.2 , 6.7)
	Q3							242.0	15.1	(12.7 , 17.7)	134.6	8.4	(6.8 , 10.2)	284.0	19.7	(16.6 , 23.1)	156.4	10.8	(8.7 , 13.3)	307.9	19.1	(16.2 , 22.2)	136.2	8.4	(6.7 , 10.4)
	Q4							267.7	19.0	(16.3 , 21.9)	91.9	6.5	(5.1 , 8.1)	308.3	22.5	(19.2 , 26.1)	138.3	10.1	(8.0 , 12.5)	355.7	19.8	(16.8 , 23.0)	167.6	9.3	(7.3 , 11.7)
	Q5							305.1	19.7	(16.7 , 22.9)	150.8	9.7	(7.4 , 12.6)	295.5	24.3	(19.2 , 30.2)	129.0	10.6	(7.6 , 14.4)	383.7	24.2	(20.4 , 28.4)	178.4	11.3	(9.0 , 13.9)
	Boys																								
**National**	**Total**							**888.0**	**11.7**	**(10.7 , 12.7)**	**379.8**	**5.0**	**(4.3 , 5.7)**	**1288.9**	**16.5**	**(15.0 , 17.9)**	**698.8**	**8.9**	**(7.9 , 9.9)**	**1366.2**	**16.3**	**(15.0 , 17.5)**	**829.9**	**9.9**	**(8.7 , 11.0)**
Area	Urban							720.2	13.7	(12.3 , 15.0)	330.5	6.3	(5.3 , 7.3)	1045.8	18.5	(16.7 , 20.4)	594.3	10.5	(9.3 , 11.8)	1108.9	17.8	(16.2 , 19.4)	703.2	11.3	(9.8 , 12.8)
	Rural							167.8	7.2	(5.9 , 8.7)	49.2	2.1	(1.6 , 2.7)	243.1	11.1	(9.3 , 13.1)	104.5	4.8	(3.7 , 6.0)	257.3	11.9	(10.3 , 13.5)	126.8	5.9	(4.7 , 7.2)
HLCI	Q1							76.7	4.6	(3.3 , 6.2)	22.3	1.3	(0.8 , 2.1)	231.3	11.7	(9.3 , 14.5)	76.5	3.9	(2.8 , 5.1)	178.1	11.0	(8.9 , 13.3)	74.2	4.6	(3.0 , 6.7)
	Q2							135.3	10.0	(8.3 , 11.8)	33.1	2.4	(1.6 , 3.5)	288.8	16.5	(13.4 , 20.0)	130.6	7.4	(5.9 , 9.3)	219.8	14.4	(12.2 , 16.8)	134.1	8.8	(6.5 , 11.6)
	Q3							197.0	12.5	(10.4 , 14.9)	86.8	5.5	(3.6 , 8.2)	242.7	16.5	(14.1 , 19.2)	145.4	9.9	(8.0 , 12.0)	276.3	16.7	(14.0 , 19.6)	173.9	10.5	(8.5 , 12.7)
	Q4							228.1	15.7	(13.2 , 18.4)	100.2	6.9	(5.3 , 8.8)	307.3	19.9	(16.8 , 23.4)	217.5	14.1	(11.0 , 17.7)	366.7	20.9	(18.0 , 24.0)	190.1	10.8	(8.7 , 13.3)
	Q5							235.2	17.3	(14.5 , 20.4)	131.5	9.7	(7.9 , 11.7)	217.1	20.4	(16.1 , 25.4)	126.1	11.9	(8.9 , 15.6)	325.2	17.7	(14.8 , 20.8)	257.6	14.0	(10.9 , 17.6)
	**Boys and girls**							**2016.5**	**13.0**	**(12.3 , 13.7)**	**835.0**	**5.4**	**(4.8 , 5.9)**	**2717.4**	**17.3**	**(16.2 , 18.3)**	**1376.8**	**8.7**	**(7.9 , 9.5)**	**2859.4**	**17.3**	**(16.3 , 18.2)**	**1481.3**	**8.9**	**(8.2 , 9.7)**
**Adolescents**	***Women***																								
**National**	**Total**	**441.5**	**7.6**	**(6.7 , 8.5)**	**97.6**	**1.7**	**(1.2 , 2.2)**	**1600.9**	**21.1**	**(19.8 , 22.4)**	**350.8**	**4.6**	**(4.0 , 5.2)**	**2018.6**	**22.0**	**(20.6 , 23.5)**	**790.6**	**8.6**	**(7.5 , 9.8)**	**1984.8**	**21.9**	**(20.3 , 23.5)**	**911.3**	**10.1**	**(8.9 , 11.2)**
Area	Urban	390.2	8.2	(7.2 , 9.2)	90.9	1.9	(1.4 , 2.5)	1225.0	22.6	(20.9 , 24.4)	286.1	5.3	(4.5 , 6.1)	1561.1	23.4	(21.6 , 25.1)	671.6	10.1	(8.7 , 11.6)	1557.5	23.1	(21.1 , 25.1)	765.5	11.4	(9.9 , 12.9)
	Rural	51.4	5.1	(3.5 , 7.3)	6.7	0.7	(0.2 , 1.7)	375.9	17.3	(15.8 , 18.9)	64.7	3.0	(2.2 , 3.8)	457.5	18.4	(15.9 , 21.1)	119.0	4.8	(3.6 , 6.2)	427.4	18.4	(16.4 , 20.6)	145.8	6.3	(5.2 , 7.5)
HLCI	Q1	73.4	6.6	(4.8 , 9.0)	2.0	0.2	(0.0 , 0.5)	224.8	16.0	(14.1 , 18.0)	13.2	0.9	(0.5 , 1.5)	327.1	16.3	(13.7 , 19.2)	90.5	4.5	(3.3 , 6.0)	279.2	18.6	(15.8 , 21.6)	95.7	6.4	(4.9 , 8.1)
	Q2	90.2	7.9	(6.0 , 10.2)	21.7	1.9	(1.0 , 3.3)	268.8	20.8	(18.0 , 23.7)	41.4	3.2	(2.5 , 4.0)	505.1	25.7	(22.8 , 28.8)	179.6	9.1	(7.1 , 11.6)	396.4	22.9	(19.7 , 26.4)	145.7	8.4	(6.7 , 10.4)
	Q3	89.9	6.8	(5.1 , 8.8)	25.4	1.9	(1.1 , 3.1)	302.5	19.4	(17.2 , 21.8)	98.9	6.4	(5.1 , 7.8)	431.0	23.3	(20.1 , 26.8)	189.1	10.2	(7.7 , 13.4)	385.1	21.7	(18.6 , 25.1)	206.4	11.6	(9.0 , 14.9)
	Q4	81.7	9.1	(7.0 , 11.8)	15.2	1.7	(0.8 , 3.3)	323.3	24.3	(21.1 , 27.6)	101.5	7.6	(5.7 , 9.9)	390.5	22.4	(19.1 , 26.1)	175.7	10.1	(7.7 , 13.0)	423.5	22.5	(19.1 , 26.1)	229.0	12.2	(9.9 , 14.7)
	Q5	81.7	7.7	(5.9 , 9.8)	29.4	2.8	(1.6 , 4.6)	448.2	24.8	(21.0 , 28.9)	91.2	5.0	(3.8 , 6.5)	359.7	23.2	(19.0 , 27.9)	151.1	9.7	(7.3 , 12.7)	500.7	23.0	(19.9 , 26.4)	234.5	10.8	(8.6 , 13.2)
	Men																								
**National**	**Total**													**1847.2**	**20.2**	**(18.6 , 21.7)**	**880.1**	**9.6**	**(8.0 , 11.3)**	**1927.0**	**20.9**	**(19.3 , 22.4)**	**1037.2**	**11.2**	**(10.1 , 12.4)**
Area	Urban													1441.7	21.0	(19.2 , 22.8)	746.8	10.9	(8.9 , 13.1)	1550.6	21.9	(20.1 , 23.8)	905.6	12.8	(11.4 , 14.2)
	Rural													405.5	17.7	(15.0 , 20.6)	133.2	5.8	(4.5 , 7.4)	376.4	17.3	(15.3 , 19.4)	131.6	6.1	(4.8 , 7.5)
HLCI	Q1													298.9	15.4	(12.7 , 18.3)	77.3	4.0	(2.8 , 5.4)	228.2	14.6	(12.0 , 17.6)	89.2	5.7	(3.8 , 8.4)
	Q2													366.5	20.0	(17.1 , 23.2)	142.9	7.8	(6.1 , 9.8)	294.1	18.6	(15.6 , 21.9)	123.7	7.8	(5.7 , 10.4)
	Q3													350.4	20.1	(17.0 , 23.6)	186.9	10.7	(8.0 , 14.1)	374.8	20.7	(17.5 , 24.1)	167.7	9.2	(7.3 , 11.4)
	Q4													400.8	22.0	(18.9 , 25.2)	218.8	12.0	(9.5 , 14.9)	499.7	24.0	(20.9 , 27.3)	314.7	15.1	(12.4 , 18.2)
	Q5													428.4	23.9	(19.8 , 28.4)	252.6	14.1	(8.6 , 22.1)	530.2	24.0	(20.3 , 28.1)	341.9	15.5	(12.8 , 18.5)
	**Men and women**													**3865.8**	**21.1**	**(20.0 , 22.2)**	**1670.7**	**9.1**	**(8.1 , 10.1)**	**3911.9**	**21.4**	**(20.2 , 22.5)**	**1948.5**	**10.6**	**(9.8 , 11.4)**

[1] Percentage and 95% confidence interval (CI).

[2] IOTF= International Obesity Task Force

[3] Overweight and Obesity definition using International cut off points for body mass index for overweight and obesity by sex between 2 and 18 years (Cole T, Bellizzi M. Establishing a standard definition for child overweight and obesity worldwide: international survey. BMJ 2000;320:1-6)

[4] Age groups defined as: preschoolers: 2-4 years; school-age children: 5-11 years; and adolescents: 12-19 years.

[5] No data available for school-age children in 1988

[6] HLCI, Household living condition index

[7] Q, Quintile
