# Supplementary material for: Overweight and obesity in Mexican children and adolescents during the last 25 years
Source: Nutr Diabetes. 2017 Mar 13;7(3):e247–. doi: 10.1038/nutd.2016.52 (PMC5380891; doi:10.1038/nutd.2016.52)
Supplement: Supplementary Figure S1 [file nutd201652x2.ppt]

## Slide 1
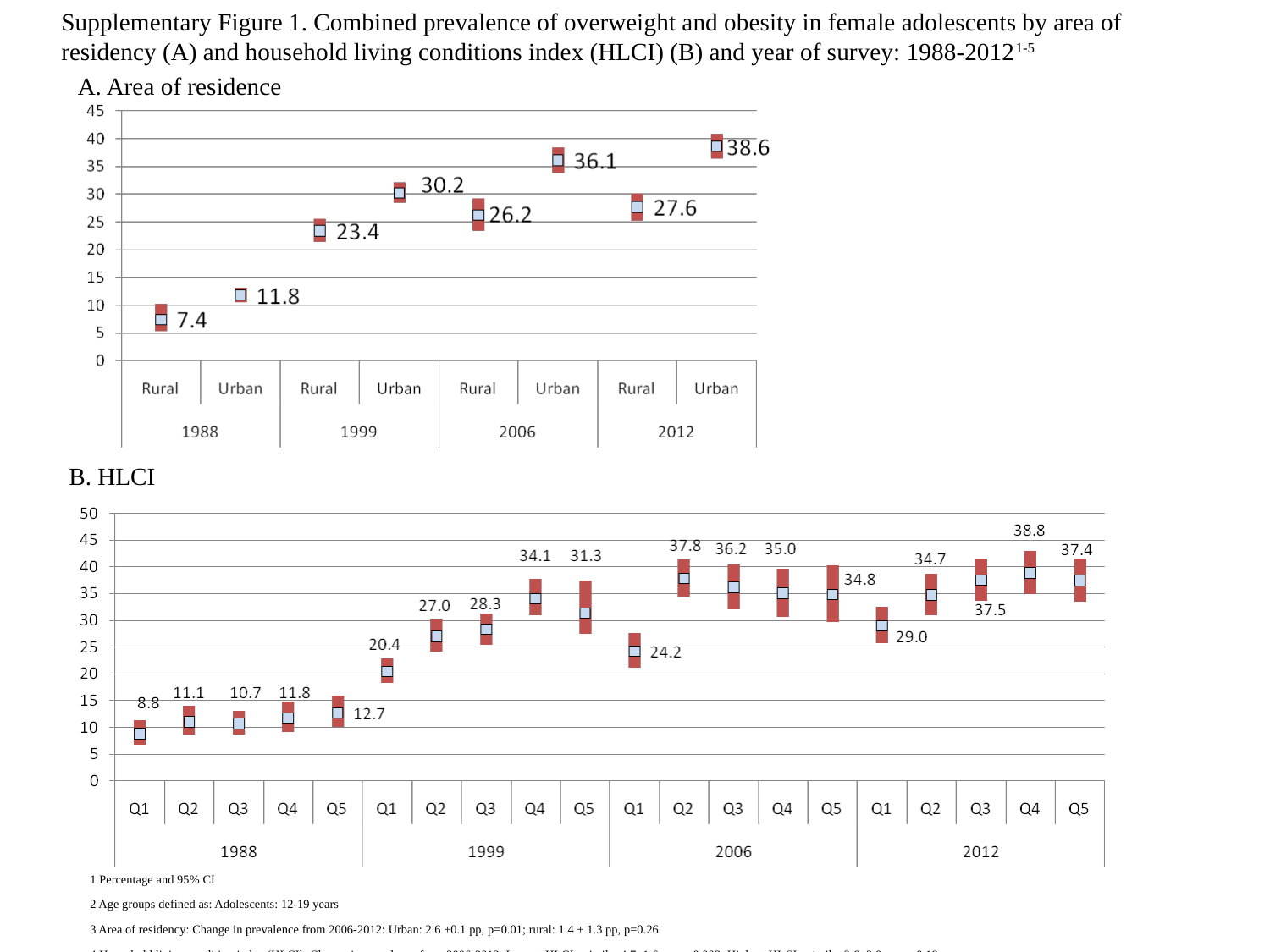

Supplementary Figure 1. Combined prevalence of overweight and obesity in female adolescents by area of residency (A) and household living conditions index (HLCI) (B) and year of survey: 1988-20121-5
A. Area of residence
B. HLCI
1 Percentage and 95% CI
2 Age groups defined as: Adolescents: 12-19 years
3 Area of residency: Change in prevalence from 2006-2012: Urban: 2.6 ±0.1 pp, p=0.01; rural: 1.4 ± 1.3 pp, p=0.26
4 Household living condition index (HLCI): Change in prevalence from 2006-2012: Lowest HLCI quintile: 4.7±1.6 pp, p=0.003; Highest HLCI quintile: 2.6±2.0 pp, p=0.18
